# Prediction of daily *PM*_2.5_ concentration in China using partial differential equations

**DOI:** 10.1371/journal.pone.0197666

**Published:** 2018-06-06

**Authors:** Yufang Wang, Haiyan Wang, Shuhua Chang, Adrian Avram

**Affiliations:** 1 Department of Statistics, Tianjin University of Finance and Economics, Tianjin, China; 2 Coordinated Innovation Center for Computable Modeling in Management Science, Tianjin University of Finance and Economics, Tianjin, China; 3 School of Mathematical and Natural Sciences, Arizona State University, Tempe, United States of America; North University of China, CHINA

## Abstract

Accurate reporting and forecasting of *PM*_2.5_ concentration are important for improving public health. In this paper, we propose a partial differential equation (PDE) model, specially, a linear diffusive equation, to describe the spatial-temporal characteristics of *PM*_2.5_ in order to make short-term prediction. We analyze the temporal and spatial patterns of a real dataset from China’s National Environmental Monitoring and validate the PDE-based model in terms of predicting the *PM*_2.5_ concentration of the next day by the former days’ history data. Our experiment results show that the PDE model is able to characterize and predict the process of *PM*_2.5_ transport. For example, for 300 continuous days of 2016, the average prediction accuracy of the PDE model over all city-regions is 93% or 83% based on different accuracy definitions. To our knowledge, this is the first attempt to use PDE-based model to study *PM*_2.5_ prediction in both temporal and spatial dimensions.

## Introduction

*PM*_2.5_ (particulate matter smaller than 2.5um), has been linked to deducing visibility, negatively affecting human’s health and impacting global climate and therefore *PM*_2.5_ pollution has aroused unprecedent public concern in the world [1]. In some places where there is critically high atmospheric pollution, government will enforce extreme actions as closing of schools and industries and restriction of motor vehicles circulation. If it were possible to predict these episodes one or two days in advance, public authorities will have more time to take more efficient actions to protect citizens, for instance, by increasing public transports in case of traffic block or by issuing an early warning to inform citizens to take preventing-*PM*_2.5_ measures.

The originate of *PM*_2.5_ is complex. Many factors such as area topography, geomorphology, emission source location and weather may have influence on *PM*_2.5_ concentration [2] and regional transported aerosols are also an important factor for *PM*_2.5_ pollution [3–5]. Similarly like stock market, *PM*_2.5_ concentrations are dynamic and exhibit wide variation, and accurate prediction of them thus becomes a highly challenging task because of the highly nonlinear nature and the complex characteristics.

Presently, studies can be divided into three groups: deterministic methods, statistical approaches and satellite remote sensing techniques. Deterministic methods, such as 3-D chemistry transport models (CTMs) [6, 7] mainly address the formation mechanism of *PM*_2.5_ from the view of physico-chemical and meteorological processes by a knowledge of the temporal dynamics of the emission quantitites of various pollutants. This kind of method needs perfect representation of the physico-chemical processes, therefore it is difficult to guarantee the real-time forecast. Statistical approaches, as empirical prediction methods, aim to detect some certain correlated patterns between air quality data and various selected predictors, thus to predict the pollutant concentrations in future. Common statistical approaches, such as linear regression models [8, 9], neural networks [10], nonlinear regression models [11, 12], neuro-fuzzy models, are easier to implement but only limited to specific geographical locations. Satellite remote sensing techniques [13] have the advantages of spatially seamless and long-term coverage, so they have been widely employed to predict *PM*_2.5_ by considering satellite-derived aerosol optical depth empirically correlated with *PM*_2.5_ in recent years, but the equipment expense is high. Each approach addresses problems from different perspectives.

Differential equation models have been extensively used in biology, sociology, economics, physics and other fields to describe various time evolution and dynamic systems. Logistic models have been widely used to describe various population dynamics and predict the growth of bacteria and tumors over time in Biology Mathematics and the spatial spread of infectious disease in epidemiology [14]. Reaction-diffusion equations describe population development in [15]. Navier-Stokes equations describe the motion of viscous fluid substances in physics [16]. In particular, leveraging geocoded data dramatically expands application of PDE models to many social problems. Recently, some researchers proposed using PDE models to forecast information diffusion in online social networks by introducing social distance [17–19].

The main contribution of this study is to introduce a PDE model of *PM*_2.5_ transport which describes the temporal and spatial characteristics of *PM*_2.5_ in China from a global view. Further we use the model to predict the next day’s *PM*_2.5_ concentrations. The PDE-based model is linear-diffusion equation built on intuitive distance metric between clusters in China. These clusters are obtained by a higher-order network analysis in [20, 21]. This is the first work to apply PDE model to predict *PM*_2.5_ concentration. As a original work, this method can help Chinese government make prediction about *PM*_2.5_ concentrations one or two days in advance.

## Methods

### Data

The research data used in this study covers 300 days from January 1st, 2016 to October 26th, 2016. The average *PM*_2.5_ daily level of each cluster is calculated from the daily *PM*_2.5_ level of all cities included in this cluster. All relevant data is available from figshare: https://figshare.com/s/ea760256b9850255eed8 (DOI: 10.6084/m9.figshare.6243254). The daily *PM*_2.5_ data can be publicly deposited from this link. Specifically, This *PM*_2.5_ data set contains 189 priority pollution monitoring cities in China’s mainland, which cover all the thirty-four provincial-level regions of China. The most polluted and most interested cities are all included, such as Beijing, Shanghai and Guangzhou. The 189 cities have been divided into 9 clusters by a higher-order complex network method, considering various weather conditions and geography conditions in [20]. The original *PM*_2.5_ concentrations of each city are normalized to a discrete level value 1, 2,…., or 6, according to “Ambient Air Quality Standards” (GB3095-1996) of China, where *PM*_2.5_ concentrations are divided into 0-35, 36-75, 76-115, 116-150, 151-250 and greater than 250 *μ*g and these different concentration ranges are leveled from 1 to 6, describing that air quality is good, mild, moderate, severe severe, highly severe or seriously severe. The linear scaling makes concentration value to a specific range, which ensures the larger value input attributes do not overwhelm smaller value inputs thus in turn helps decrease prediction errors.

## Clustering and embedding

**Fig 1:**
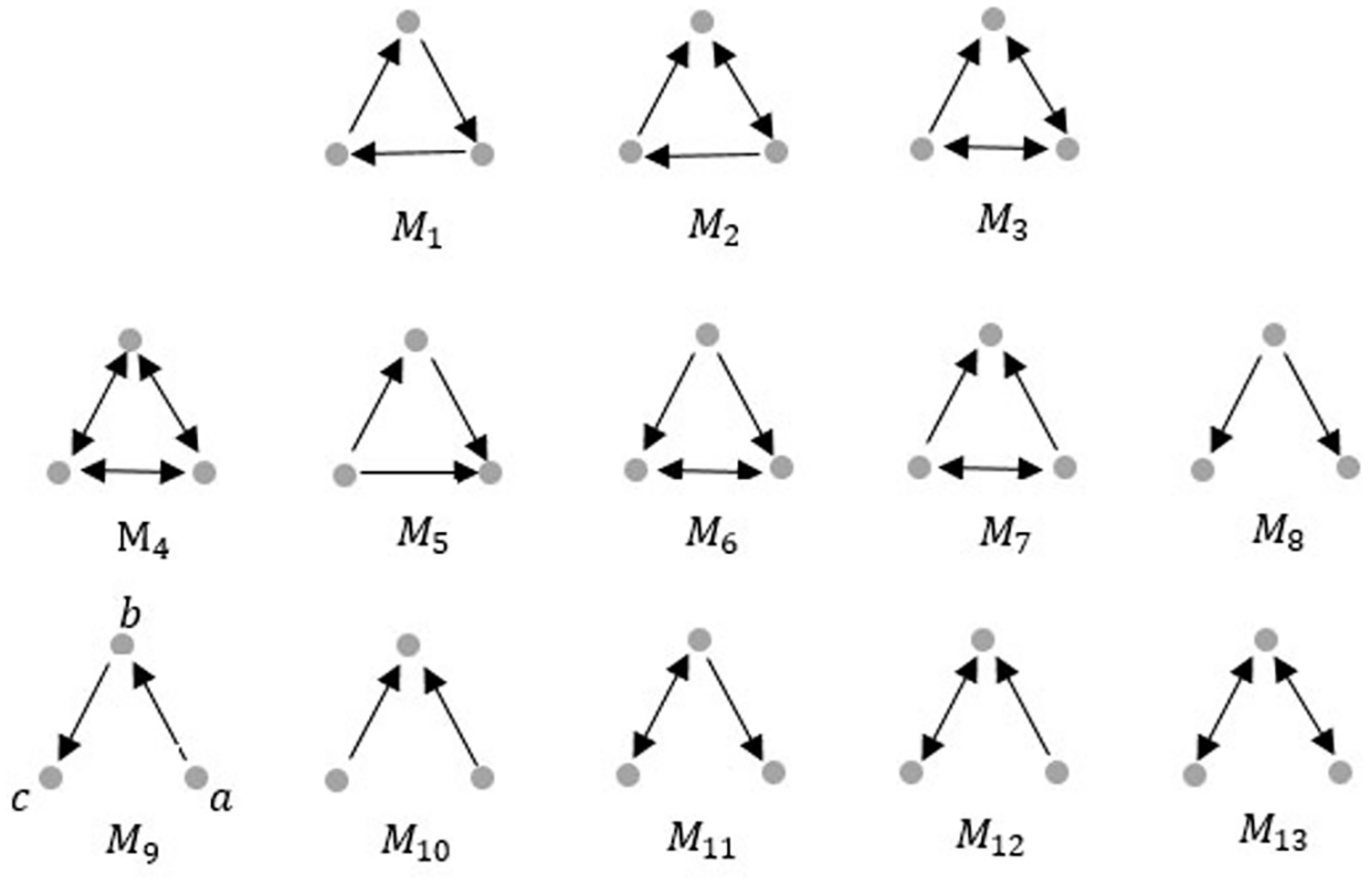
Triangle motifs.

**Fig 2:**
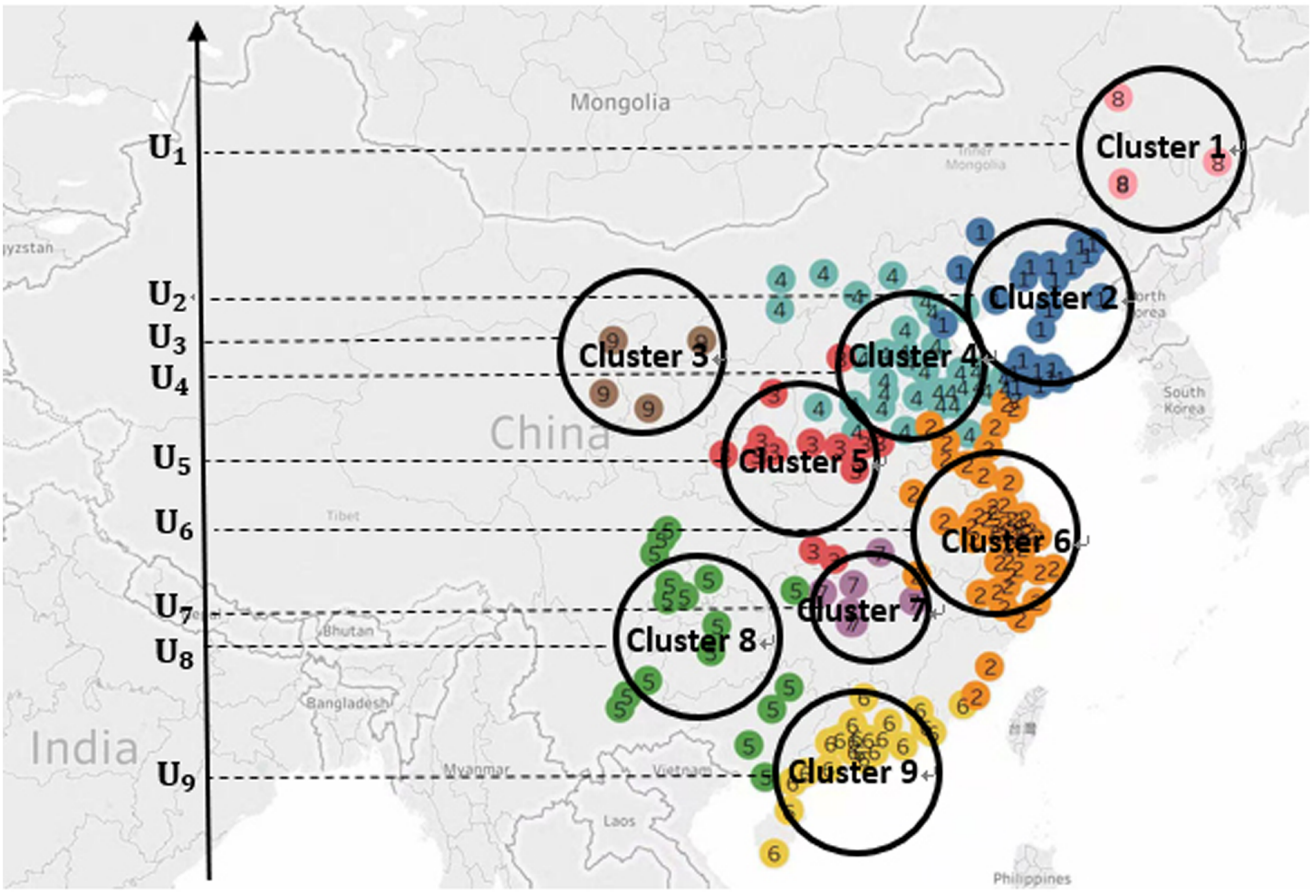
Nine clusters obtained by M_8_-motif spectral clustering algorithm [20]. Tableau Public 10.3 (https://public.tableau.com/) was used to create the map.

**Fig 3:**
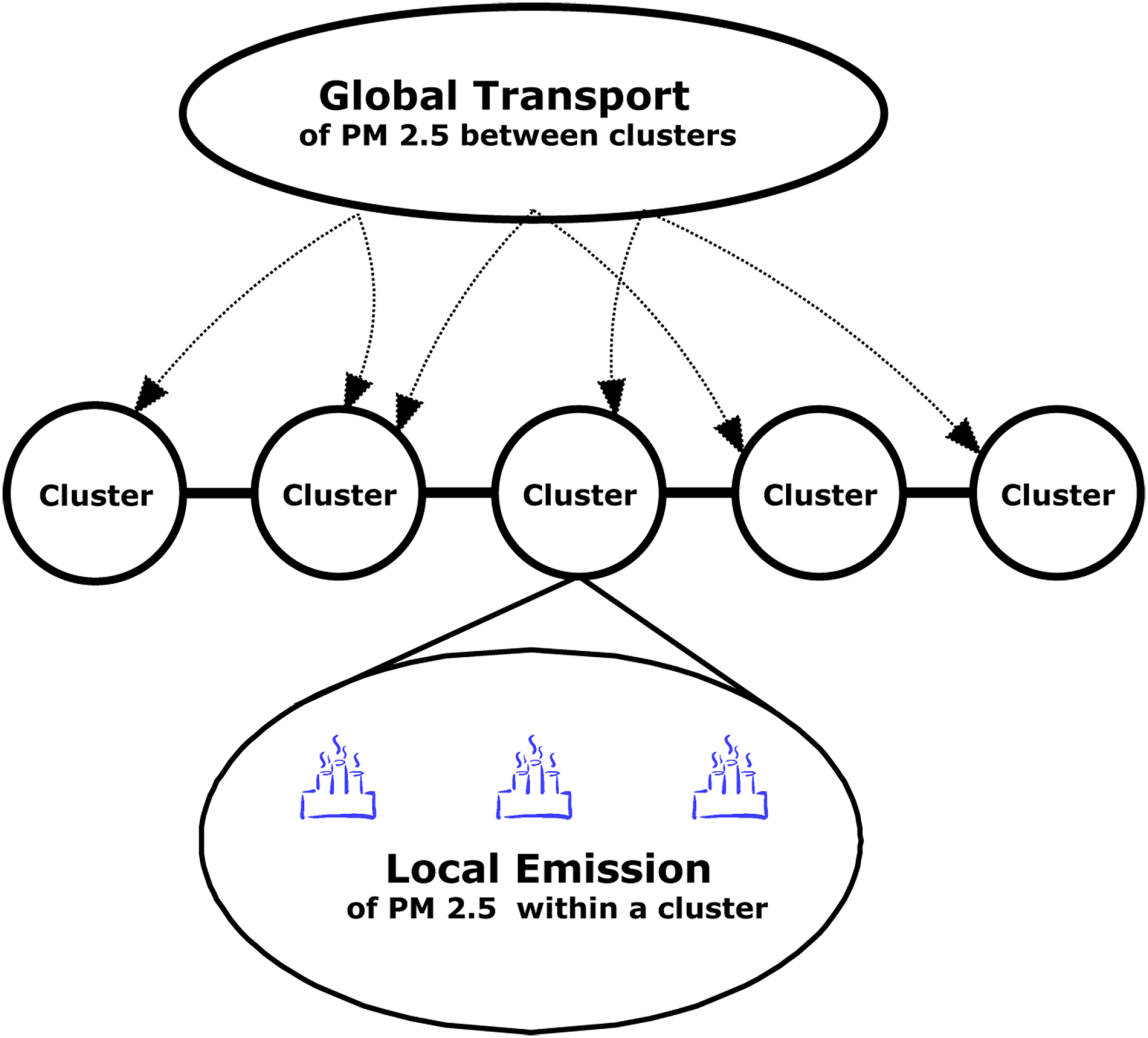
PM_2.5_ transport processes.

In our previous work [20], we have divided 189 main cities into 9 clusters by a higher-order spectral method. Specifically, the higher order organization, motif *M*_8_ in Fig 1, reflects the *PM*_2.5_ movements from source to target in *PM*_2.5_-city network. We applied motif *M*_8_ as the basic building block of complex network and applied the higher-order spectral method in [22] to divide 189 main cities of China into 9 clusters in Fig 2. Cities in each cluster are more affected with each other about *PM*_2.5_ pollution (local emission) and *PM*_2.5_ of cities among different clusters also possibly influences each other through air flow (regional transport). Therefore in this paper we propose a partial differential equation in spatio-temporal dimension to describe the dynamic system of *PM*_2.5_ transport. Similarly as information diffusion on social network, for a city cluster, one part of its *PM*_2.5_ is produced by itself from its road dust, vehicle exhaust, industrial emission, agriculture activities, which can be seen as local emission. The other part of its *PM*_2.5_ is from other city clusters through regional transport [3–5]. Therefore, the change of *PM*_2.5_ concentration in a city cluster equals the amount from regional transport plus the *PM*_2.5_ produced by the city itself (Fig 3).

As we demonstrate in the last paragraph, we could divide 189 cities of China into 9 clusters, i.e., *U* = {*U*_1_, *U*_2_, …, *U*_9_} through a higher-order spectral clustering method [20, 22]. Once the entire group of 189 cities are partitioned into disjoint sets *U*_*i*_, *i* = 1, 2, …, 9, we will order *U*_*i*_ in a meaningful way. For general clustering partition, the spatial arrangement of *U*_*i*_ can be based on specific modeling goal and social or geographical characteristics of the underlying network. In [23, 24], the level of democracy, diaspora size, international economic relations and geographical proximity are used to order *U*_*i*_. In [17], friendship hops are used to define distance metric, then *U*_*x*_ is embed at location *x* based on that *x*-axis is used as the social distance. For *PM*_2.5_ transport in China, ordering cluster *U*_*i*_ by geographical characteristics and meteorological characteristics are the best intuitive method. Wind is a main factor influencing air transport [2, 25, 26] and China is with a monsoon. Specifically, in about September the winter monsoon first reaches northern China and soon moves southly to the Yangtze River and the South China Sea. Summer monsoon prevails to southern China in early May; Then it prevails to the southwestern region in late May or early June; Mid-summer monsoon suddenly prevails into the Yangtze River and Huaihe River Basin in June, mid-summer monsoon again moves to North China and Northeast China in July. Therefore the monsoon of China leads to the fact that most of its wind directions are south-to-north or north-to-south. Therefore, It is meaningful to project the 9 city clusters located from north to south in the map of China onto y-axis of the Cartesian coordinates, and name these city clusters as *U*_1_ to *U*_9_ as their geographical location from north to south as in Fig 2.

These clusters are naturally embedded into the two dimensional Euclidean space. As a result, the PDE model, which describes *PM*_2.5_ transport between the clusters, would be two-dimensional PDEs. For simplicity, in this paper, we project the clusters into y-axis and focus on one dimensional PDE. We will study two-dimensional PDE models in a future work.

### Linear PDE model

Differential equation can describe lots of dynamic systems in fields of physics, mathematical biology, social science and many other fields [27–30], particularly the “information” diffusion problems. In epidemic models, it describes the spread of infectious diseases among a population [29, 31] to help people understand the disease’s spreading patterns, such as trends and ratio of people infected. Epidemic models have inspired many unsolved mathematical questions and re-energized novel mathematical researches [27]. In recent century, epidemic models also have been adopted to describe and predict computer virus infections [32, 33]. Recently, some works [17–19]use differential equations to describe information propagation such as news and rumors on social network.

Ordinary differential equation(ODE) and partial differential equation(PDE) are two basic and main classes of models to describe dynamic models. The former is more suitable for studying global patterns, such as trends of people getting infected by epidemics, but cannot describe the connections among individuals, such as who infects whom. However, besides time variable *t*, partial differential equation has spatial factors involved. Therefore, spatial dimension provides a quantitative way to describe the local interactions between individuals of the system into global views.

PDE models obey the energy conservation law [34] that is the rate at which a quantity changes in a given domain must equal the rate at which it flows to the boundary plus the rate at which it is created or destroyed in the domain. In different fields, conversation law can describe different scenarios [17–19]. In the following, we propose a linear PDE-based model to abstractly translate the *PM*_2.5_ transport in two processes: local emission and global transport (in Fig 3).

To quantify the conversation law for *PM*_2.5_ concentration, let *q*(*y*, *t*) denote the average *PM*_2.5_ concentration of the cities in the cluster at distance *y* during time *t*. The value of *q*(*y*, *t*) depends on two processes. First, the cities in *U*_*y*_ can produce *PM*_2.5_ by themselves and we call this process as local emission. Secondly, *PM*_2.5_ in *U*_*x*_ where *x* ≠ *y* can flow into *U*_*y*_ by wind, namely regional transport [2] and we call this regional transport as global transport.

Following is the description of the PDE model

$$\begin{array}{c} \begin{array}{c} \frac{\partial q(x,t)}{\partial t}=\frac{\partial }{\partial x}(d\left(x\right)\frac{\partial q(x,t)}{\partial x})+r\left(t\right)q\left(x,t\right)h\left(x\right),\\ q(x,1)=\phi (x),l<x<L,\\ \frac{\partial q}{\partial x}\left(l,t\right)=\frac{\partial q}{\partial x}\left(L,t\right)=0,t>1, \end{array} \end{array}$$

where

- $\frac{\partial }{\partial x}(d\left(x\right)\frac{\partial q(x,t)}{\partial x})$ is the global transport, which describes the regional transport of *PM*_2.5_ between different clusters. This mathematical expression is widely used in spatial biology and epidemiology [30], where it describes the spatial spread of infectious disease.
$-d\left(x\right)\frac{\partial q(x,t)}{\partial x}$ measures the amount of the quantity crossing the section at *x* and time *t* and this results from a principle analogous to Fick law [34].
  - *d*(*x*) is a positive function of *x* and it describes the *PM*_2.5_ transport ability of the cluster at location *x*. Actually, different regions in China have different *PM*_2.5_ transport abilities, therefore using a positive piece-wise function to describe *d*(*x*) will be better, which will be studied in our future work. To be simple, we use $d(x)=e^{-b_{0}x}$ in this paper, here *b*_0_ is a parameter which needs to be determined by the real data.
- *r*(*t*)*q*(*x*, *t*)*h*(*x*) represents the local emission and it denotes the rate at which *PM*_2.5_ is produced or dissipated within the cluster at location *x* and time *t*. The amount of reproduction or dissipation is proportional to both the existing amount of *PM*_2.5_ and the amount of various varying resources. This mathematical expression has been used to describe and predict various population dynamics such as the growth of bacteria and tumor over time [34].
  - h(x) represents the spatial heterogeneity of *PM*_2.5_ in the cluster at location *x* and it depicts the *PM*_2.5_ emissions from various sources such as road dust, vehicle exhaust, biomass burning, industrial emissions of this city cluster. In this paper, different clusters represent different regions in China. Different levels of economy, population of different regions lead to essentially different *PM*_2.5_ emissions [2]. *h*(*x*) is constructed by piecewise first-order Lagrange Interpolation with the interpolated points are *x* = 1, 2, …, 9, where each cluster is located, therefore nine references *b*_*i*_ = *h*(*x*_*i*_), *i* = 1, 2, …, 9 need to be determined.
  - *r*(*t*) is the growth rate with time *t* in local process. It depicts *PM*_2.5_ dissipation with the external changing factors such as wind or certain other atmospheric conditions [2, 25, 26]. Without reproduction, *PM*_2.5_ will dissipate with wind, therefore the form *r*(*t*) can be expressed as *r*(*t*) = *α* + *e*^−*β*(*t*−*δ*)^, where *α*, *β* and *δ* are constant values. This form of *r*(*t*) has also been used in describing information decay in online social network [17]. More plausible expression of *r*(*t*) will be discussion in our future work.
- $\frac{\partial q}{\partial x}\left(l,t\right)=\frac{\partial q}{\partial x}\left(L,t\right)=0,t>1$, is the Neumann boundary condition [34]. To be simple, here we suppose there is no flux of *PM*_2.5_ across the boundaries at *x* = *l*, *L*.
- *q*(*x*, 1) = *ϕ*(*x*) is an initial *PM*_2.5_ concentration function, which can be constructed from the history data of *PM*_2.5_ by the cubic splines interpolation.

The basic mathematical properties, such as existence, uniqueness and positivity of the solution of the models can be established from the standard theorems for parabolic PDEs [35]. In this paper, the PDE is validated with real data from China National Environmental Monitoring Center.

### Accuracy definition

We evaluate the performance of the proposed linear diffusive model by comparing the concentration level calculated by the model with the actual observations. *y*_*t*_ denotes the actual value, and ${\hat{y}}_{t}$ is the predicted value. Then the prediction performance is evaluated from two different technical indicators.

- MAID (mean absolute increment accuracy), which is proposed in this paper based on the practical significance, defined as
  $$\text{MAID}=\frac{1}{n}\sum_{t=1}^{n}\text{AID}(\text{t}),$$
  where
  $$\text{AID}(\text{t})=1-\frac{|y_{t}-{\hat{y}}_{t}|}{5},$$
  *n* is the number of sample points in test data set and AID evaluates the absolute accuracy at each sample point. There are totally five *PM*_2.5_ concentration levels from level one to level five and AID describes the absolute accuracy in the view of level length. For example, if $|y_{t}-{\hat{y}}_{t}|=0.6$, then the prediction accuracy of this sample point is $1-\frac{0.6}{5}=0.88$.
- MRA(mean relative accuacy), which is defined as
  $$\text{MRA}=\frac{1}{n}\sum_{t=1}^{n}(1-\frac{|y_{t}-{\hat{y}}_{t}|}{y_{t}}),$$
  where *n* is the number of the sample points in the test data set.

## Results

After we finish the work of collecting the real *PM*_2.5_ concentration data, we conclude the prediction process as follows: Firstly, we normalize the data for decreasing the experimental errors and calculate the average *PM*_2.5_ daily level of each cluster. Then, we use the training data set of three days to predict the *PM*_2.5_ concentration for the following day, i.e., we use days 1-3, 2-4, 3-5,…as training data, and predict the following day 4, 5, 6,…correspondingly and record the prediction accuracy for all 9 regions at the day 4, 5, 6,…. Specifically, we give the detailed prediction process for day 4 as an instance. For the training data set of day 1, 2, 3, we interpolate the discrete data of day 1 in constructing the initial function *ϕ*(*x*) of the PDE model; Next, we use the data of day 2 and day 3 to train the parameters of the PDE model by fminsearch function in matlab; Finally, we use this PDE model with a new initial function *ϕ*(*x*) trained by the data of day 3 to predict the *PM*_2.5_ concentration of day 4.

**Fig 4:**
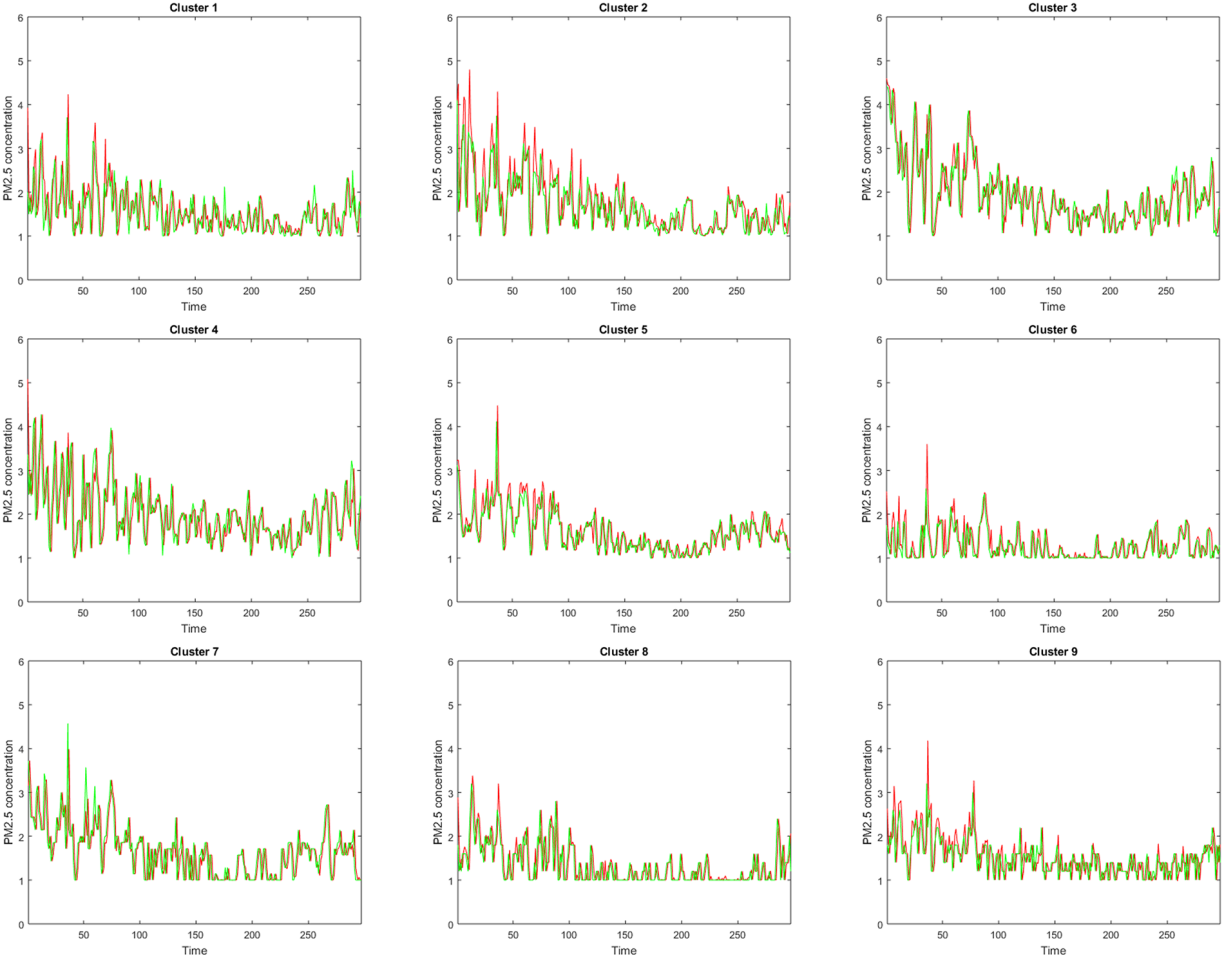
The daily PM_2.5_ concentration level from January 4, 2016 to October 26, 2016. Red line represents the prediction value and green line stands for the real data.

**Fig 5:**
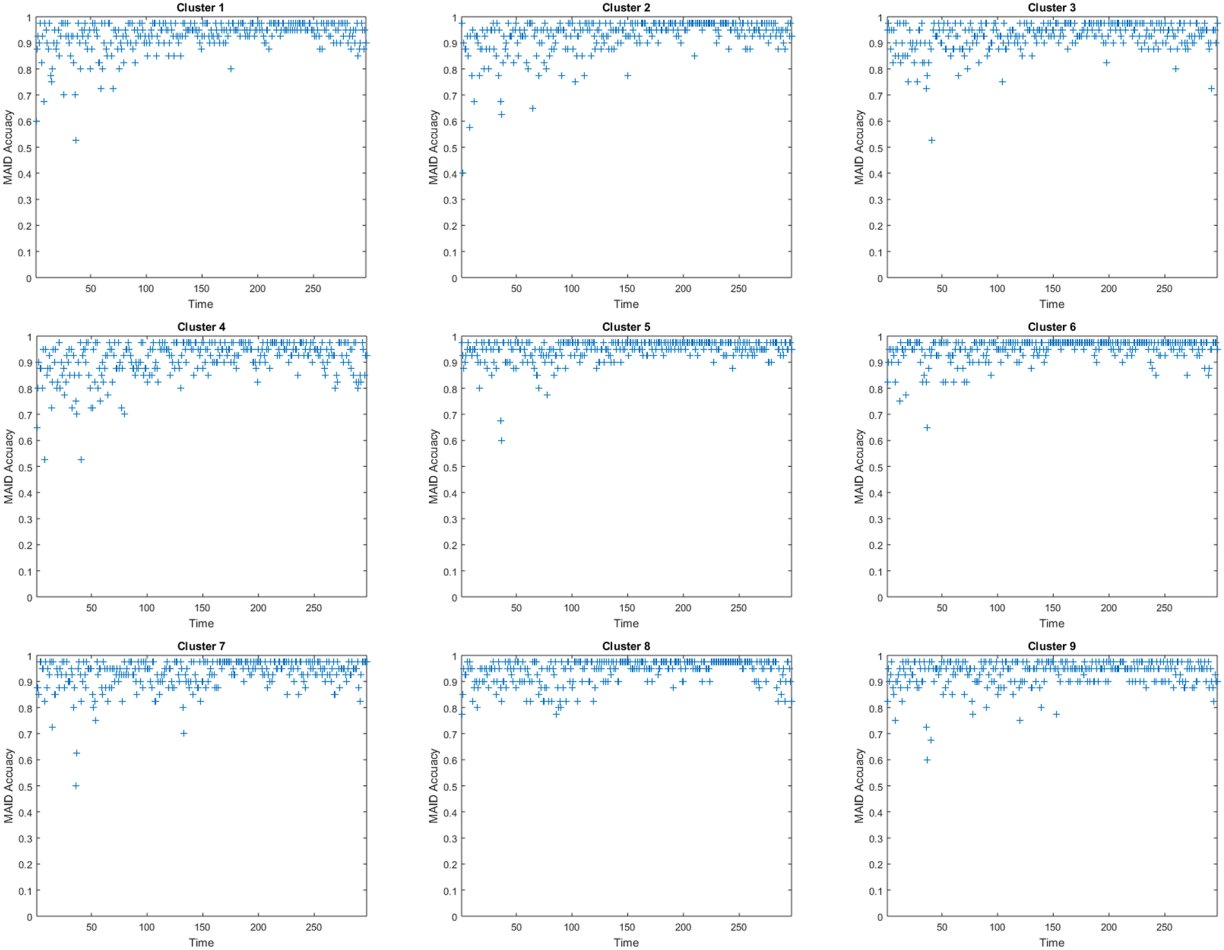
The accuracy of each cluster (city region) from January 4, 2016 to October 26, 2016, based on the accuracy definition of MAID.

**Fig 6:**
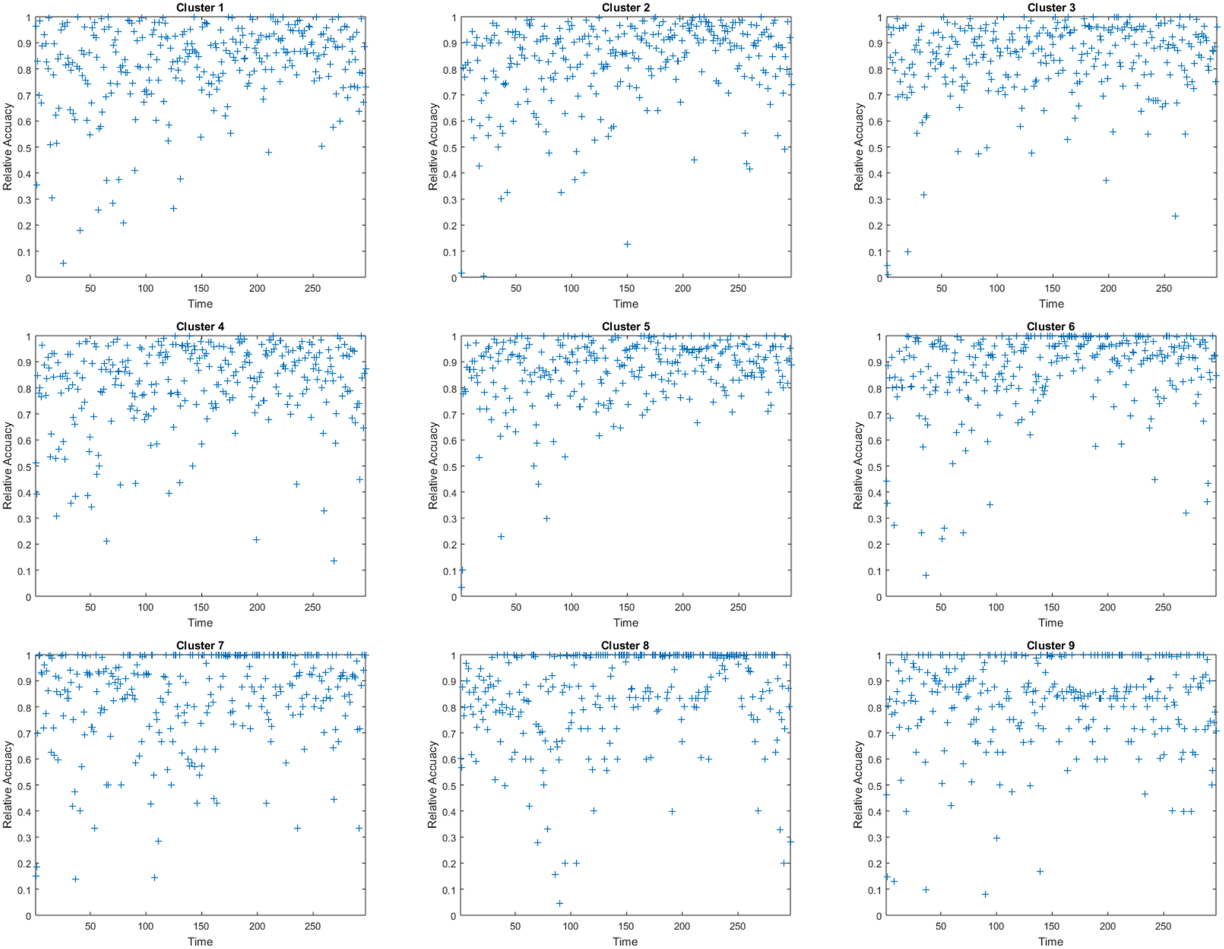
The accuracy of each cluster (city region) from January 4, 2016 to October 26, 2016, based on the accuracy definition of MRA.

Fig 4 illustrates the prediction results of regions 1-9 at each day from January 4, 2016 to October 26, 2016. Clearly to see that there is a consistent trend between predicted data (represented by red lines) and real observations (presented by green lines). Specifically, Figs 5 and 6 show the prediction accuracy of each city region at different time *t* over 300 days from January 4, 2016 to October 26, 2016. Based on the accuracy definition MAID, in Fig 5 most of plus marks exist above the horizontal line of 0.9, which means that most of days have a 90% higher prediction value for each cluster and in Table 1 the mean accuracy values of the 9 clusters are all higher than 91%. In Fig 6, the plus marks are not so gathered like that in Fig 5, that is because the accuracy definition of MRA is a rule which measures the accuracy of *PM*_2.5_ concentration value while MAID is a rule which measures the accuracy of *PM*_2.5_ concentration value range. In Fig 6, we can still find that most of plus marks exist above the horizontal line of 0.8, which means that most of days have a 80% higher prediction value for each cluster and in Table 1 the mean accuracy values of the 9 clusters are all higher than 80%. Besides, we also find that in Table 1 the average prediction accuracy of the PDE model over all city regions and over all days is 93% and 83% based on the two different accuracy definition respectively.

**Table 1:** The average accuracy of different regions and the average accuracy over all regions based on different accuracy definition.

	MAID	MRA		MAID	MRA
Region 1	0.926178451	0.806965014	Region 6	0.948400673	0.858288077
Region 2	0.926262626	0.821313941	Region 7	0.928367003	0.834495023
Region 3	0.924410774	0.823766934	Region 8	0.939478114	0.848018478
Region 4	0.911363636	0.802610969	Region 9	0.930808081	0.809905853
Region 5	0.946212121	0.865824998	Regions 1-9	0.93127572	0.830132143

**Fig 7:**
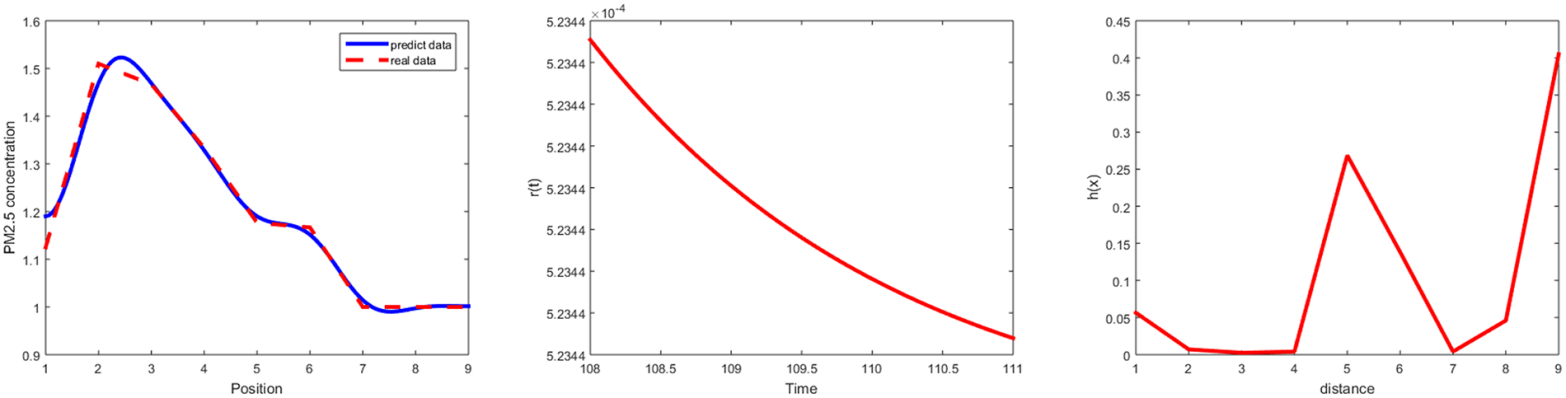
The predicted results of each cluster, h(x) and r(t) of the PDE model on April 27, 2016.

Specifically, Fig 7 lists the predicted results and the graphs of *h*(*x*) and *r*(*t*) on April 27, 2016 as an instance. The parameters in *h*(*x*) and *r*(*t*) on April 27, 2016 are listed in Table 2. The results of other days are omitted due to space limit.

**Table 2:** The values of these parameters in h(x) and r(t) of the PDE model on April 27, 2016.

Parameter	Value	Parameter	Value	Parameter	Value
*b*_1_	0.031296298	*b*_5_	0.125161344	*b*_9_	0.211384796
*b*_2_	0.430962739	*b*_6_	0.096457084	*α*	0.000000025
*b*_3_	0.009605521	*b*_7_	0.000040027	*β*	0.001943744
*b*_4_	0.000081749	*b*_8_	0.054862183	*δ*	7.245630868

## Discussion

In this paper, we propose a PDE-based model to characterize *PM*_2.5_ transport in 189 main cities of China in order to make short-term prediction of *PM*_2.5_ concentration. This is an innovative try to use mathematical methods to describe air pollution problem. This work aims to help Chinese government or health organization to forecast the *PM*_2.5_ concentration one or two days in advance and raise an alarm for specific regions at these moments when there is a high probability that the *PM*_2.5_ concentration is high.

However, the prediction performance and the PDE model may be improved further in the following respects. Firstly, We can embed the problem into a high dimension space *R*^2^ by considering a few other variables that may affect the prediction performance. To better describe *PM*_2.5_ transport, robin boundary condition or others will also be discussed in our future work. A piecewise positive function about the diffusion term *b*(*x*) will better describe the reality and we will perfect it work in the following work. Secondly, in this paper, we choose the increment between each cluster to be 1, it can be adjusted according to the specific cases. Certainly, a better increment is based on a more better distance metric of the system. Lastly, we could propose an surveillance system based on the prediction outcomes of this study for practical use.
